# An in vitro study of lipid preference in whaleworm (*Anisakis simplex*, Nematoda, Ascaridoidea, Anisakidae) third-stage larvae

**DOI:** 10.1007/s00436-013-3748-x

**Published:** 2014-01-24

**Authors:** Einar Strømnes

**Affiliations:** Natural History Museum, University of Oslo, P.O.Box 1172 Blindern, NO-0318 Oslo, Norway

## Abstract

The behavioural response of nematodes to chemical stimuli has been extensively investigated in some free-living and plant parasitic species. However, in animal parasitic species, little is yet known, particularly in regards to marine forms such as the whaleworm (*Anisakis simplex*). Previous studies showed that *A. simplex* L3-larvae tend to prefer fish tissue with high lipid content. The intention of this study was to investigate the behaviour of *A. simplex* L3 in response to different concentrations of fish lipid in further detail. This was done by an in vitro study based on larvae from cod (*Gadus morhua*). Ten larvae were placed in each of the culture containers containing agar that was separated into three segments of equal size. Three categories of agar were used containing 0, 2 and 7 % cod liver oil. A total of 900 larvae were included. The study consisted of three parts: The purpose of experiment I was to establish whether different lipid concentrations influenced the migration pattern at all. Experiment II was intended to examine whether *A. simplex* L3-larvae were able to actively search for lipids. Experiment III was set up to analyse the short-distance dispersion of the L3-larvae. Experiment I indicated that the L3-larvae move randomly but do not *stop* randomly since the tendency to move out of the start area was inversely correlated with lipid concentration. Experiment II indicates that the larvae are almost unable to select areas of high lipid concentrations when more than a few centimetres away. Experiment III showed that the L3-larvae prefer high-fat content and can seek it out over short distances.

## Introduction

Even though the presence of *Anisakis simplex* larvae in fish flesh cause considerable year-on-year economic losses in the fishing industry, little is known about the chemical mechanisms governing the movement of the larvae in the fish hosts. In nematodes, behaviour in relation to chemical stimuli has been extensively studied in some free-living species such as *Caenorhabditis elegans* and certain species of plant parasites. In these cases, it appears that the amphids are the primary organs involved in responses based on smell or taste (Riga et al. 1995). Far less is known regarding responses to chemical stimuli in animal parasitic nematodes and most studies have been of species in which the larvae actively penetrate the skin. Such larvae are also capable of following gradients, and can register a potential host by means of both chemical and thermal stimuli, as well as mechano-sensorial stimuli (Granzer and Haas 1991; Ashton et al. 1999).

Regarding marine animal parasitic species, even less is known about their response patterns in connection to chemical conditions. However, the general impression in studies dealing with the distribution of *A. simplex* in fish and cephalopods is that they, to a large extent, spread in a random manner throughout these hosts (e.g., Novotny and Uzmann 1960; Wootten and Waddell 1977; Smith 1984; Manfredi et al. 2000).

Young (1972) suggested that *A. simplex* L3-larvae do not orient themselves according to chemical stimuli, but that the distribution in fish is a result of a random “optimal pre-encapsulation migratory distance”. However, this hypothesis was not supported by Strømnes and Andersen (1998). On the contrary, this study showed that L3-larvae had a certain preference for tissue with high lipid content, whereas the distance from the intestine (the point of entry) was of less importance. In order to further elucidate this issue, the questions of whether the observations made in wild fish could be verified under experimental conditions were addressed in the present study.

As opposed to free-living nematodes and the larval stages of terrestrial plant and animal parasitic nematodes, the larvae of *A. simplex* are spread randomly throughout the marine food web without any influence over which host they enter (e.g., Anderson 2000). Hence, for parasites with this type of life cycle, it is important to be highly flexible in their way of life. A goal-seeking behaviour in terms of localising a new host is, therefore, presumably of little adaptive relevance. Strømnes and Andersen (2003) found that the length of *A. simplex* L3 was significantly correlated with host tissue fat content. Even though it is evolutionarily rational for *A. simplex* L3 to be a generalist, this may indicate that it is an adaptive advantage to be oriented towards encapsulation in a more fat-rich host tissue.

*Anisakis simplex* L3 can be found throughout many fish hosts, in all kinds of tissues (Wootten 1978; Smith 1984; Bahool et al. 2012). Nevertheless, Strømnes and Andersen (1998) showed more larvae in lipid-rich fish tissue. Thus, larval distribution may be governed by a behaviour based on random migration coupled with non-random stopping due to some biochemical signal. An evident candidate for a stopping cue is fish lipids. Consequently, it is expected that the L3-larvae would show a greater tendency to stop at higher lipid concentrations resulting in a positive correlation between larval abundance and lipid content of the micro-environment. In short, the aim of the present study was to investigate whether *A. simplex* L3-larvae have the ability to react behaviourally to variations in lipid concentration.

## Materials and methods

Initially, a pilot in vitro study was performed. Here, the capacity for movement in *A. simplex* L3-larvae turned out to be relatively low: no larva moved more than 10.5 cm from its starting point. In addition, most of the movement took place within 3–4 days. Based on these results, an experiment with 12 × 12-cm culture dishes and a duration of 2 weeks was taken to give a representative picture of the movement pattern of the larvae in question.

The *A. simplex* L3-larvae used in this study were collected from four specimens of cod (*Gadus morhua* L.), 80–85-cm long, caught outside Eggum in the Lofoten archipelago in northern Norway (68º22′N, 13º38′E) in mid-June of 2002. A sub-sample was collected by first picking medium-sized larvae (about 2.5 cm) from the cod livers. These nematodes were put into a 250-ml standard laboratory beaker containing 150 ml of physiological saline solution (distilled water containing 0.9 % NaCl). Subsequently, a pair of tweezers was employed to randomly pick up the pre-decided number of specimens. The larvae were kept in a solution of Pepsin A (Fluka, CAS-reg. no. 9001-75-6) and HCl (Smith and Wootten 1975), and larvae were left in this solution for 15 min at 20 ºC to soften the host tissue surrounding them and possibly giving them a stimulus to move. Finally, the remaining host tissue was removed.

An agar solution of 0.7 % (Swelling index 13, Norwegian Medicinal Depot) was found to be optimal regarding the density of the medium. A greater density seemed to inhibit the larvae’s moving capability, while a lower content of agar resulted in a culture medium that was too liquid. The agar mixture was based on distilled physiological saline solution.

**Fig. 1:**
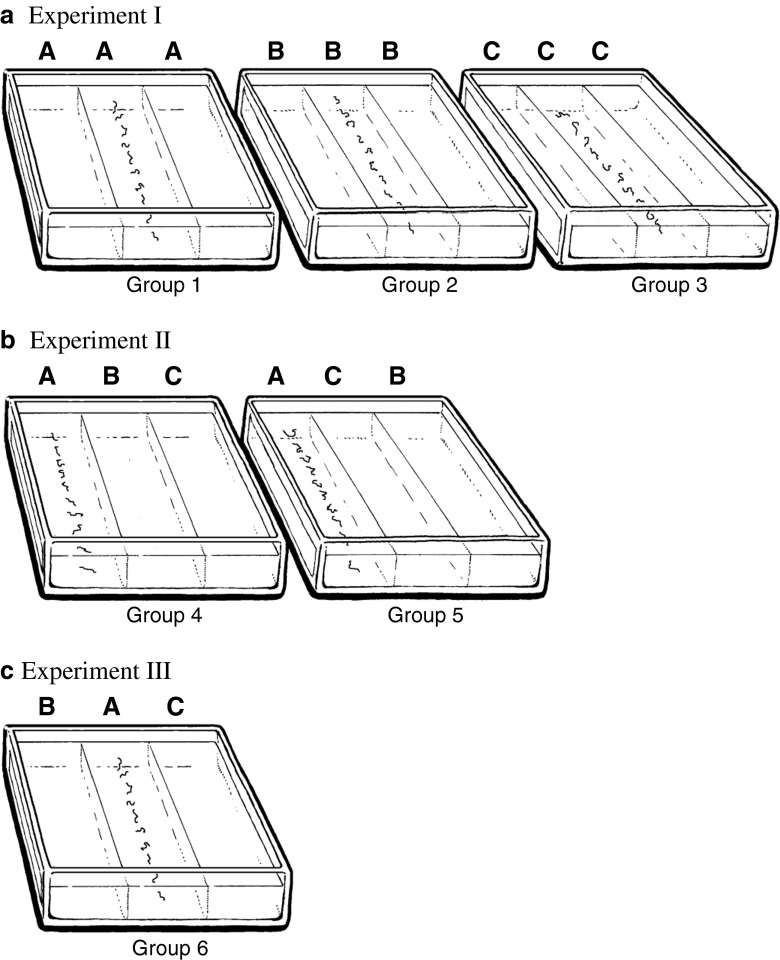
**a**, **b**, **c** The experimental designs: culture dishes with three segments of agar and the position of the *Anisakis simplex* L3-larvae (short irregular lines) at the start of the experiments. The L3-larvae were implanted 1-cm below the surface and maintained in darkness at 12 ºC for 14 days. Three categories of agar were used: *A* = 0 % oil; *B* = 2 % oil and *C* = 7 % oil. Experiment I: The dishes were divided into three groups (*groups 1*, *2* and *3*), each with one agar category only, either *A*, *B* or *C*. The L3-larvae were implanted along the centre line of the middle segment. Experiment II: The dishes were divided into two groups (*groups 4* and *5*). The L3-larvae were in the oil-free A segment at one end of the dish, while the positions of segments B and C were varied. **c** Experiment III: Consisted of one group (*group 6*). The larvae were, as in experiment I, implanted along the centre line of the middle segment

Three categories of agar were used (Fig. 1): A: agar based on physiological saline solution only (oil-free, i.e. 0 % cod liver oil); B: agar with 2 % and C: agar with 7 % standardised cod liver oil (“Møllers™ tran”, Norway) added. A maximum value of 7 % was selected since this was found to be the approximate “saturation point” for this type of oil in the agar. However, even at this value, some oil was exuded during the experimental period. An oil concentration of 2 % was selected as a reasonable estimate of a low concentration relative to 7 %.

In wild fish, the lipid content of tissues can vary greatly throughout the year depending on temperature and conditions of nourishment. In cod (from where the larvae in this experiment were collected), the liver is the most lipid-rich organ and may contain more than 30 % lipids (Love 1988). This is far above the “saturation point” of the agar used in the present study, and is one reason why it is difficult to relate the current in vitro environment directly to the in vivo environment of the larvae. Hence, the relationship between these two conditions will not be further addressed in this study.

All the culture dishes in the study were filled with a 1.8–2.0-cm layer of agar (see below) and divided into three equal-size segments, each 4-cm wide. Ten L3-larvae were then implanted 1-cm below the surface in the beginning segments. The agar layer consisted of clearly delineated segments, rather than gradients, since this, to a greater degree, mirrors the categorically organised micro-environment in the fish host organs. In an attempt to approximate the thermal conditions in fish from the sampling area at the current time of the year (Institute of Marine Research, Norway, http://www.imr.no/forskning/forskningsdata/stasjoner/), the culture dishes were placed in a room with a constant temperature of 12 °C and darkness. At the end of the experimental period, the culture dishes were placed on a light table, and the distribution pattern of the nematodes was registered.

The investigation was divided into three parts (Fig. 1). In order to investigate whether lipid concentration differences influence the movement pattern of L3-larvae, one experiment (experiment I) was designed with three groups of culture dishes, each containing only *one* of the three categories of agar (A, B or C). If *A. simplex* L3-larvae do not respond to cod liver oil as a chemical cue, one would expect a random distribution in all three culture groups. In the following, the group of culture dishes containing only oil-free agar, category A, is designated as *group 1*, the group with only agar of category B as *group 2*, and the group with only agar of category C as *group 3*.

**Fig. 2:**
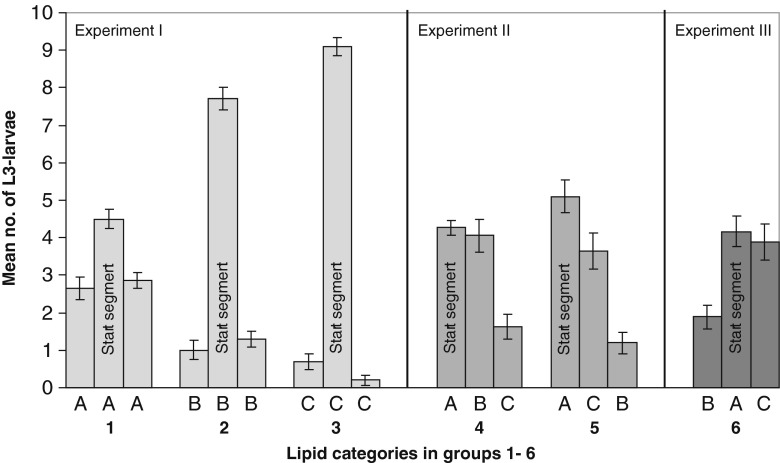
Mean numbers of *Anisakis simplex* L3 larvae in the agar segments from the six culture groups by the end of the study. Experiment I (*light grey columns*), where the L3 larvae were implanted along the centre line of the middle segment in all the dishes. Experiment II (*medium grey*) and experiment III (*dark grey*), where the L3 larvae were placed along the centre line of the oil-free A segment. *A* = 0 % oil, *B* = 2 % oil and *C* = 7 % oil. *Error bars* represent ±standard error (SE)

Although the agar for experiment I was made in one batch, it was cut into three equally wide segments after hardening in the dishes in order to create conditions as similar as possible to the conditions in experiments II and III, where each culture dish contained all three categories (A, B and C) of agar (see below and Figs. 1 and 2). In experiment I, the *start segment* was always the *middle* segment. Here, and throughout the study, the larvae were placed along the centre line of this segment. Experiment I was repeated with ten parallels and a total of 300 larvae.

In order to investigate whether *A. simplex* L3-larvae are capable of seeking out the most lipid-rich microhabitats available, experiment II contained *two* different culture groups (Fig. 1b). In both groups, the starting point for the larvae was the oil-free A segment, localised at one end of the culture dishes. Then, the relative positions of the oil-containing segments were switched, so that in one group, the low-concentration segment B bordered segment A. On the other hand, in *group 5*, the high-concentration segment C bordered A. This gave the segment sequence A B C and A C B for *groups 4* and *5*, respectively. In experiment II, the L3-larvae were placed along the centre line of the A segment.

If L3-larvae are capable of seeking out the most lipid-rich microhabitat within their radius of action, in experiment II, one should expect that a higher proportion of larvae would be localised in the C segment compared to the B segment regardless of the different distances to the C segment, *groups 4* and *5*, respectively. In contrast, if the larvae do not have the capacity to respond to different oil concentrations, one would expect a distribution corresponding to that of experiment I, *group 1*.

To achieve additional information about the lipid-seeking behaviour of the L3-larvae, a third experiment (experiment III) was designed: The starting segment A was placed in the *centre* of the culture dishes, with segments B and C on either side. Thus, the distance factor that had been introduced in experiment II was eliminated. This group of culture dishes (segment order B A C), will subsequently be designated as *group 6*. Here, the L3-larvae were again placed along the centre line of the oil-free segment A. If L3-larvae prefer a high concentration of lipids, one would expect that more larvae in *group 6* migrated to segment C than to segment B.

Experiments II and III were repeated with 20 parallels since the variance in these experimental designs were greater than in experiment I. Thus, experiment II contained 400 larvae, while experiment III (i.e. *group 6*) contained 200 *A. simplex* L3-larvae.

To prepare the cultures containing agar with different oil concentrations (experiments II and III), the dishes were first filled with the oil-free agar (category A). The A section was then cut away to make room for a segment of category B agar. Once this mixture had hardened, the last section was cut away and filled with category C agar. In an attempt to reduce seeping of exuded oil to bordering segments, the oil-free A segments were made 2-cm thick, while the B and C segments were 1.9- and 1.8-cm thick, respectively.

To determine whether the number of L3-larvae was significantly different in the various segments, both within a group and between the groups 1–6, the data were analysed using the general linear models with a binomial distribution (S-plus). In those cases where the larvae started from the middle segment, the non-parametric Wilcoxon paired sample test (ANOVA) was used since this test is more robust than a paired *t* test.

## Results

The distribution of L3-larvae at the end of the experiment is summarised in Fig. 2. Only one dead larva (in experiment II) was registered at the end of the study.

For *group 1* (i.e. 0 % oil in the entire culture), an average of 4.5 out of 10 larvae were localised in the starting segment at the end of the experiment. The two bordering segments in *group 1* both contained an average of three larvae. The culture dishes in *group 2* (i.e. 2 % oil in the entire culture) clearly had more larvae in the starting segment (7.7), and an average of about 1 larva in the bordering segments. In *group 3* (i.e. 7 % oil in the entire culture), very few larvae in this most lipid-rich environment had moved away—or had relocated and moved back—from their starting segment. Concerning *group 3*, an average of 9.1 specimens was localised in the starting segment in the middle of the culture dish by the end of the experiment. The number of L3 found in the starting segments of experiment I was significantly different (deviance = 65.53, *DF* = 2, *p* < 0.001). Even the results from *groups 2* and *3*, showing the least prominent distinction, were significantly different (deviance = 7.51, *DF* = 1, *p* = 0.006).

In experiment II, the start segment was positioned at one end of the culture dishes. *Group 4*, with the segment order A B C, showed a decreasing number of larvae with increasing distance from start segment A. The average number of larvae remaining in segment A was 4.3. The average number of larvae in the bordering segment B was 4.05 L3, and in the C segment, at the most distant end of the dish, it was 1.63 L3.

The distribution in *group 5* (A C B) showed a similar pattern as that of *group 4*, with a decreasing number of L3 from the starting segment towards the far end of the dish. *Group 5* had more larvae in the starting segment (5.1), but somewhat fewer in the bordering C segment (3.65) and the most distant B segment (1.2).

These differences in *groups 4* and *5* were not significant (ANOVA, *p* > 0.20). There was, however, a tendency to a greater concentration of larvae in segment A, *group 5* than in *group 4* (deviance = 2.79, *DF* = 1, *p* = 0.094). The number of larvae in segment B, *group 4* was significantly higher than in the left and right segments in *group 1* (*p* = 0.003). The number of larvae in the C segment, *group 4* was significantly less than in the left and right segments in *group 1*. These differences were also found to be significant with *p* = 0.005 and *p* = 0.002, respectively.

In experiment III (*group 6*, B A C), where the distance from the starting segment to each of the oil-containing segments (2 and 7 %) was equal, a significantly higher number of L3-larvae had migrated to the C segment than to the B segment (*p* = 0.007, one-tailed Wilcoxon signed-rank test).

## Discussion

Among the few papers addressing the question of mechanisms behind the distribution pattern in marine parasitic nematodes L3-larvae in fish, Young (1972) proposed a hypothesis of an “optimal pre-encapsulation migratory distance”. The suggestion here is that the infestation of anisakid larvae in fish muscle (where they cause a medical problem) would decrease relative to the infestation in the viscera as the size of the fish increases, since the distance from the digestive tract to the muscle tissue would consequently increase. However, Strømnes and Andersen (1998) found that the distribution between tissues was relatively independent of the fish size, and that the distribution pattern also varied considerably among the three species studied (cod, saithe and redfish). On the other hand, these variations seemed connected with the distribution of lipids in the various host species, indicating that this matter required a more elaborate explanation than that of a random and distance-based migration within the host. A plausible explanation might be that the distribution is influenced by the chemical conditions of the microhabitat where ample access to lipids is preferred.

One factor supporting this explanation is the correspondence found by Strømnes and Andersen (2003) between the lipid content of three tissue categories, muscle, liver, other viscera and the size of the *A. simplex* larvae. The L3-larvae found in tissue with high levels of lipids seemed to exhibit a faster growth rate than larvae found at lower lipid levels. If the size of the larvae is presumed advantageous for survival, this may also indicate that a certain capability to actively seeking nutrient-rich microhabitats is beneficial to the species in an evolutionary sense.

The results from experiment I indicate that larval mobility diminished as the oil concentration increased. In a situation where the microhabitat contains little or no lipids, as in *group 1*, the L3-larvae are apparently stimulated to increase their mobility and actively seek new and possibly better microhabitats. As access to lipids increases, as in *groups 2* and *3*, the need to seek microhabitats with higher levels of lipids seems to be diminished, and fewer larvae are triggered to move away from their start segment.

In all six combinations of segments, a considerable percentage of the larvae did not move away from their starting segment. The size of the seemingly immobile fraction remaining in the starting segment was constant (45–50 %) in all the groups containing oil-free segments (*groups 1* and *4*–*6*). The larvae were not monitored systematically during the study period, so at least some of the specimens may have been visiting the neighbouring segments and then had returned to the starting segment.

By the end of the experiment, there was an approximately equal number of larvae in the starting segment and in the bordering B segment in *group 4* (segment order A B C), while the number of larvae in the C segment was significantly lower. If *A. simplex* L3-larvae are not capable of goal-seeking behaviour, the distance involved, with the starting segment and the C segment at opposite ends of the dish, could offer an explanation for this result.

In *group 5* (A C B), the L3-larvae demonstrated a distribution pattern largely congruent with the pattern in *group 4*. However, there was a tendency in the material to find more L3-larvae in the A segment in *group 5* than in *group 4*. Assuming that the oil to some extent diffuses from one segment to another, this observed difference between *groups 4* and *5* may be a reasonable result. The larvae in the start segment in *group 5* would have access to more oil than those in *group 4*, since the A segment in *group 5* borders on the oil-rich C segment, while the A segment in *group 4* borders on the less oil-rich B segment.

In *group* 6 (B A C), where the start segment was localised in the middle of the culture dishes, distance was largely eliminated as a variable. Here, significantly more larvae had moved to the C segment and then to the B segment. This tendency of more larvae preferring the microhabitat with the highest lipid content is expected if *A. simplex*-L3 have a preference for oil, and do not only move randomly from their start point.

The results from experiment I, thus, demonstrate that an increasing concentration of fish oil will reduce the *A. simplex* L3-larvae’s tendency to move. In other words, an oil deficit will stimulate migrating behaviour, while this inclination gets weaker at higher oil concentrations as the stop signal gets stronger. Experiment II further indicates that the larvae were not able to examine the entire area available and choose the most favourable segment within the period of time covered by this study.

Nevertheless, experiment III shows that if the larvae, when placed in an environment with little or no fat, are given a choice between areas with different concentrations of fat, they do tend to prefer the most oil-rich environment. These factors indicate that *A. simplex* L3-larvae move randomly, but do not *stop* randomly, since their tendency to migrate is increasingly inhibited, or their inclination to stop is incited, as the concentration of oil increases.

In experiment I, the distribution pattern observed in *group 1* exhibited a random distribution of L3, which could be explained if the migrating larvae moved with random speed and direction, without being stimulated to stop. Similarly, it may be suggested that the distribution in *groups 2* and *3* can be explained by an increasing number of larvae experiencing a sufficient inhibition of their inclination to move with an increasing concentration of lipids. Regardless of the actual mechanism, the net migration distance will decrease.

In experiment II, *group 4*, the percentage of L3 in the starting segment was approximately equal to the A segment in experiment I, *group 1*, and the reason for this appears to be the same. In the bordering B segment, there were an approximately equal number of larvae as found in the A segment. This number was also significantly higher than in the segments bordering the A segment in *group 1*, while the number in the C segment was significantly lower than both. According to the present hypothesis this is a reasonable result. As migrating L3-larvae arrive in segment B, the oil concentration increases, and the probability of the larvae stopping, increases. In this way, the larvae would accumulate in segment B. This also implies that an oil concentration of 2 % is sufficient to make the presumed inhibiting effect clearly noticeable. However, although the inclination may be decreased, some larvae may manage to reach the more distant C segment, where the probability of migration inhibition is even greater. One indication of this may be that in *group 4*, where the most distant segment contains the most oil (C); there are more larvae than in the most distant segment in *group 5*, even if this difference is not significant. In *Group 5*, the mechanisms will probably be of a similar nature. In this group, slightly fewer larvae move to the most distant segment, and additionally, fewer move out of their starting segment.

In the single group of experiment III (*group 6*), significantly more larvae were found in the most oil-rich segment C than in the less oil-rich segment B. The observed distribution lends support to the process described by the present hypothesis. Those larvae that through random movement arrive in segment B, will probably have their inclination to move inhibited, and thus they will stop or reduce their velocity. Larvae that do not stop completely, might to some extent, move back to the starting segment and from there over to segment C. There, the oil concentration is so high that, as shown in *group 3*, the probability of further movement becomes very low. For those larvae that by chance arrived first in segment C, there is a correspondingly small probability of further movement. In this way, more larvae might accumulate in segment C than in segment B.

The present study supports that *A. simplex* L3-larvae, in accordance with Young (1972), radiate randomly out from the digestive tract of the fish host. However, contrary to Young’s optimal pre-encapsulation migratory distance hypothesis, the *A. simplex* L3-larvae seem to actively relate to their chemical environment by, at the very least, sensing the fat concentration in their habitat. This suggests a tendency towards reduced migration at higher levels of oil concentration implying an increased probability of L3-larvae accumulating in high-fat tissues of the host. This non-random distribution of *A. simplex* L3-larvae corresponds to findings in wild fish outlined in the introduction (Strømnes and Andersen 1998). This is also in accordance with Sukhdeo and Sukhdeo (1994, 1997) who found that endoparasites, living in a very predictable environment like the inside of a fish host, orient themselves by use of chemical cues in a fixed action pattern and not by following gradients.
